# Tele-neurology in Latin America: digital solutions for a treatment gap

**DOI:** 10.3389/fpubh.2026.1779415

**Published:** 2026-02-20

**Authors:** Jose E. Leon-Rojas

**Affiliations:** 1 Escuela de Medicina, Universidad de Las Américas, Quito, Ecuador; 2 Grupo de Investigación Bienestar, Salud y Sociedad, Escuela de Psicología y Educación, Universidad de Las Américas, Quito, Ecuador

**Keywords:** LATAM, project ECHO, TeleEEG, teleneurology, telestroke networks

## Abstract

Neurological disorders remain a leading cause of disability across Latin America, yet access to specialist care is affected by important workforce shortages, geographic disparities, and under-resourced health systems. Tele-neurology has emerged as a promising strategy to mitigate these barriers, particularly in the wake of the COVID-19 pandemic, which resulted in rapid digital health adoption. This review article examines the development and implementation of tele-neurology initiatives across Latin America, with a focus on Ecuador; drawing on examples such as TeleEEG, telestroke networks, and Project ECHO, I illustrate how digital tools have expanded the reach of neurological services in underserved regions. Despite demonstrable benefits, challenges persist, including uneven digital infrastructure, regulatory gaps, and disparities in access. I argue that tele-neurology must be deliberately integrated into national public health strategies, not merely as a pandemic contingency but as a potential long-term solution for health equity, if done properly. Strategic investments in broadband access, clinician training, sustainable financing, and regional collaboration are essential to scale these innovations. When anchored in strong policy frameworks and aligned with global neurological health goals, tele-neurology could offer a path toward closing the treatment gap and advancing equitable neurological care throughout Latin America.

## 1 Introduction

Latin America faces a substantial neurological care gap driven by a low density of specialists and significant urban–rural disparities; many countries in the region have fewer than 1 neurologist per 100,000 potential patients, far below the 5 per 100,000 considered as adequate by the World Health Organization (WHO) (1–3). Moreover, those neurologists are heavily concentrated in capital cities and large urban centers, leaving rural and remote communities with minimal or no specialist access (1, 4, 5). For example, in Brazil the vast Amazon region has long had the fewest neurologists, reflecting a broader pattern of maldistribution that exacerbates existing healthcare inequities (5). The shortage is compounded by limited training opportunities for new neurologists; for instance, in Argentina only 0.67% of all residency positions in 2023 were allocated to neurology, forcing many aspiring specialists to train abroad (4). Lastly, in Ecuador, a 2023 analysis reported that there are 0.54 neurologists per 100,000 individuals with the vast majority (39.4%) located in the capital city, Quito, and in the main port Guayaquil (25.5%); with some provinces in Ecuador having no neurology specialists at all (1). This workforce deficit directly translates into unmet needs for patients with neurological disorders across Latin America, with evident treatment gaps for common neurological conditions. Epilepsy, for instance, illustrates this issue; only about 40–50% of people with epilepsy in Latin America receive appropriate treatment, meaning over half go untreated and in rural areas, the epilepsy treatment gap may reach as high as 80–90% (6–10). Similar care gaps likely affect other chronic neurological disorders such as Parkinson’s disease, stroke, and multiple sclerosis (11–13). Patients in underserved regions often face delayed diagnoses, unmanaged symptoms, and higher disability rates due to lack of timely specialist care (11–13). These disparities highlight the urgent need for innovative approaches, beyond the traditional model of in-person neurologist consultations, to deliver neurological expertise to the populations that remain out of reach.

## 2 The emergence of tele-neurology during COVID-19

Even before 2020, telemedicine was recognized as a tool to expand healthcare access in Latin America, but adoption in neurology was modest. The COVID-19 pandemic proved to be a turning point that accelerated tele-health implementation; lockdowns and social distancing measures in 2020 forced healthcare systems to embrace digital health solutions virtually overnight (14, 15). Telemedicine quickly became a necessary component of clinical practice for both COVID-19 cases and routine care, allowing continuity of services while minimizing infection risk (15). In the neurological field specifically, providers and patients turned to tele-neurology (remote neurology consultations via video or phone) to ensure ongoing care for conditions like epilepsy, stroke, and movement disorders when clinics were closed (16, 17). In the first half of 2020, the global public health emergency catalyzed widespread adoption of tele-neurology as neurologists sought to preserve access to care (18). Notably, tele-neurology had seen only limited uptake prior to the pandemic, but the crisis rapidly increased acceptance among specialists and health authorities, establishing tele-neurology as a potentially viable care model (17, 18). Providers who may have been skeptical about virtual neurologic exams became more comfortable managing patients via video, and many patients, even those in remote areas, gained exposure to receiving neurologic care remotely for the first time. Early reports from the pandemic’s initial phase indicated that tele-neurology was largely effective for outpatient management and was well-received by many patients and families, with preliminary outcomes suggesting comparable quality of care in certain contexts (17, 19).

Additionally, the pandemic spurred regulatory and policy changes that enabled tele-health. Emergency measures in many Latin American countries allowed telemedicine practice and reimbursement where previously it was restricted; for example, Ecuador, Chile, and Uruguay had developed legal frameworks to regulate tele-health usage, which facilitated the rapid scale-up during COVID-19 (20). The experience demonstrated that many neurological consultations (for example, follow-ups for seizure control, migraine, or Parkinson’s disease medication adjustments) could be effectively conducted through telemedicine; patients benefitted by avoiding long travel to urban centers, and healthcare systems realized tele-neurology’s potential to extend specialist reach (21–23). However, the pandemic also highlighted challenges. In both global and Latin American settings, a “digital divide” emerged; meaning that some patient groups had more difficulty accessing tele-neurology than others (24–26). Lower-income, older adults, and rural patients often faced barriers such as lack of internet connectivity or devices and lower digital literacy, leading to lower uptake of video consultations (18, 26). For example, a cohort study that included 148,402 participants in the United States, reported that older age, Asian race, language, and Medicaid were related with less fulfilled telemedicine consultations; furthermore, older age, female sex, Black race, Latin-American descent, and lower household income were related to the use of less video in telemedicine consultations (24). In response, many providers resorted to telephone calls when video was not feasible, underscoring that tele-neurology is not a one-size-fits-all modality. As the acute phase of the pandemic subsided, the central question became how to sustain and integrate tele-neurology into routine health care, maintaining the progress made, while addressing the gaps and inequities that were observed.

## 3 Examples of tele-neurology innovations in Latin America

Multiple tele-neurology models have emerged across Latin America, illustrating how digital tools can help bridge the aforementioned specialist gap. Table 1 summarizes selected programs and initiatives, highlighting their scope, modalities, and significance in reducing disparities.

**Table 1:** Selected tele-neurology initiatives in Latin America, illustrating various modalities and their roles in mitigating the specialist treatment gap.

Program/Model (Country)	Scope and goals	Modalities used	Relevance/impact
TeleEEG Program (Ecuador)	Remote epilepsy diagnosis in underserved areas (e.g., Amazon basin and Galápagos). Launch: 2022 (Tena, Ecuador) (27)	Low-cost EEG devices in rural clinics; EEG data uploaded to cloud; volunteer neurologists abroad interpret EEGs via web (27)	Enabled around 20,000 EEG readings over 10 years across 20 countries, improving epilepsy diagnosis and treatment in communities with no local neurologist. Example of international tele-neurology collaboration (28).
Telestroke Networks (Argentina, Brazil, Chile, Colombia, Ecuador, Mexico, Paraguay, Peru)	Acute stroke management via telemedicine. Connects smaller hospitals to stroke neurologists for rapid thrombolysis decisions (29).	Videoconferencing for emergency neuro consult; real-time CT image sharing; telephone backup.	Increased thrombolytic therapy and improved stroke outcomes in remote hospitals. Several Latin American countries have established telestroke services, though coverage remains limited compared to high-income countries (29). These networks demonstrate life-saving potential of tele-neurology in acute care.
Project ECHO Neurology (Regional)	Capacity-building and specialist support through telementoring. Aims to “move knowledge, not patients” by training primary care providers to manage neurological cases.	Regular virtual case conferences (via Zoom/Teams) between hub (neurologists) and spoke sites (rural clinicians); case-based learning and mentorship (30).	Operating in >20 countries in Latin America (notably for epilepsy and mental health), ECHO networks have improved provider knowledge and confidence in treating neurological conditions locally (30). Facilitates task-sharing by linking general practitioners with specialist guidance, extending care to isolated areas.
Telerehabilitation for Neurologic Recovery (Multiple)	Remote therapy services for stroke, spinal injury, and Parkinson’s patients unable to access in-person rehab. Expanded during COVID-19 to maintain continuity of care.	Video-based physiotherapy and speech therapy sessions; mobile apps for exercise routines; telemonitoring of progress.	Enabled continued rehabilitation during lockdowns (26) and reached patients in rural locales with no rehab facilities. Early reports from Brazil, Peru, and Colombia showed improved adherence to therapy through telerehab services, though challenges with internet access were noted (26). This model addresses the long-term care gap after neurological injuries.
Latin American Brain Tumor Board	Regional virtual tumor board for complex neurosurgical cases (31). Facilitates multi-expert review of patient cases across countries.	Monthly teleconference with neurosurgeons, neurologists, oncologists from multiple countries; image and pathology data sharing via secure platforms.	Allows pooling of specialist expertise in neuro-oncology across Latin America (26, 31). Smaller countries without certain subspecialists can get input on patient management. Enhances clinical decision-making and regional collaboration through telemedicine.

One notable example is TeleEEG in Ecuador, a program demonstrating how international collaboration can bring specialty diagnostics to remote areas; it launched in 2022 at a regional hospital in Tena (in the Ecuadorian Amazon), equiping rural clinics with affordable EEG machines and connecting them to an online platform where volunteer neurologists worldwide interpret the EEG recordings (27). This enables patients in under-resourced communities, including indigenous areas and even the Galápagos Islands, to receive an accurate epilepsy diagnosis without traveling to a distant city. Over the past decade, the broader TeleEEG network (a UK-based charity initiative) has facilitated over 20,000 EEG interpretations in 20 countries via cloud-based telemedicine, translating relatively small investments in equipment into over £1.5 million worth of medical services provided by specialists remotely (28). The Ecuador experience with TeleEEG highlights tele-neurology’s power to reduce the epilepsy treatment gap by reaching patients who previously had no access to diagnostic tests or neurologist expertise.

Given that stroke is a leading cause of death and disability in Latin America, but timely interventions like thrombolytic therapy are often unavailable outside major urban hospitals; another emerging model is telestroke, connecting general hospitals to stroke neurologists through telemedicine (29). Countries such as Chile and Brazil have piloted telestroke networks that link smaller provincial hospitals with on-call stroke specialists via video (32, 33). In these programs, when a patient with acute stroke presents to a hospital, the local team can initiate a teleconsultation with a neurologist who reviews the patient remotely (including CT scans) and guides the administration of thrombolysis treatment if appropriate (32, 33). Early data from global surveys show that Latin America and the Caribbean have begun establishing such networks (at least 16 telestroke networks identified region-wide) (29). While most telestroke services globally still reside in high-income countries, the recent increase in Latin American telestroke initiatives is a promising development (29). These networks have shown their validity; for instance, Chile reported a similar use of thrombolysis before and during the COVID-19 pandemic, as well as faster decision times with telestroke support, likely translating to better patient outcomes (33). However, the challenge remains to scale these pilots into national systems so that stroke care is not limited by geography.

Latin America has also seen creative uses of mobile and digital tools in neurology care delivery. During the pandemic, WhatsApp and other messaging apps became informal telemedicine channels, with neurologists using them to check in on patients, adjust medications, or review images when official telehealth platforms were not available (34). Recognizing the ubiquity of mobile phones, some health systems are developing more secure mHealth applications for patient follow-up; for example, smartphone apps for seizure tracking in people with epilepsy, or SMS-based reminders for patients with memory disorders (35–37). In Argentina and Mexico, pilot programs have tested smartphone-based Parkinson’s disease monitoring (using sensors in wearable technology and/or smartphones) to allow specialists to remotely assess patients’ tremor and gait information (38). While these initiatives are in early stages, they represent a significant opportunity; mobile health can penetrate areas where traditional telemedicine is scarce, utilizing cellular networks to maintain a link between patients and providers. Additionally, the rise of artificial intelligence (AI) in healthcare is beginning to influence tele-neurology; AI-driven diagnostic tools, such as algorithms to read EEGs or MRI scans, can be integrated with telemedicine platforms to assist general doctors in interpreting results when a neurologist is not immediately available. For example, in Brazil the national Center for Artificial Intelligence has promoted AI adoption in imaging analysis, which could aid remote hospitals in reading brain scans (39, 40). More broadly, experts note that combining AI with telemedicine can improve efficiency and expand access because AI can automate routine tasks, triage referrals, and generate clinical decision support, thereby reducing the burden on limited specialist staff and allowing them to focus on patient care (41). An illustrative case is Uruguay’s longstanding investment in an interoperable electronic health record (EHR) system; it has created a unified platform where telemedicine consultations (including neurology) and AI tools can interface with patient data across the country (42). This digital infrastructure lays the groundwork for advanced tele-neurology applications, from tele-consults to population-level neurology surveillance.

Finally, the region has also embraced tele-education and task-sharing models like Project ECHO to amplify specialist impact. Project ECHO (Extension for Community Healthcare Outcomes) uses a telementoring approach where specialist teams (hubs) regularly meet via videoconference with primary care providers in rural or underserved areas (spokes) for case-based discussions and training (30). In Latin America, by 2025 there are ECHO networks in 20 countries addressing gaps in fields such as epilepsy, mental health, and chronic diseases (30). For neurology, ECHO programs have allowed general practitioners and even community health workers to manage conditions like epilepsy or headache with remote guidance from neurologists. This kind of tele-neurology network builds local capacity and creates a community of practice that can sustain care in areas without resident neurologists. For instance, a neurology ECHO in Central America has primary doctors present challenging epilepsy cases via Zoom, receiving input on diagnosis and treatment adjustments from an epilepsy specialist panel, thus enabling patients to be treated in their hometown clinics. Such initiatives align with broader public health strategies of task-shifting, wherein basic neurological care is delivered by non-specialists with specialist support; not only do they increase access, but they also reduce professional isolation and improve providers’ confidence in managing neurological disorders.

## 4 Barriers to implementing and sustaining tele-neurology

**Figure 1:**
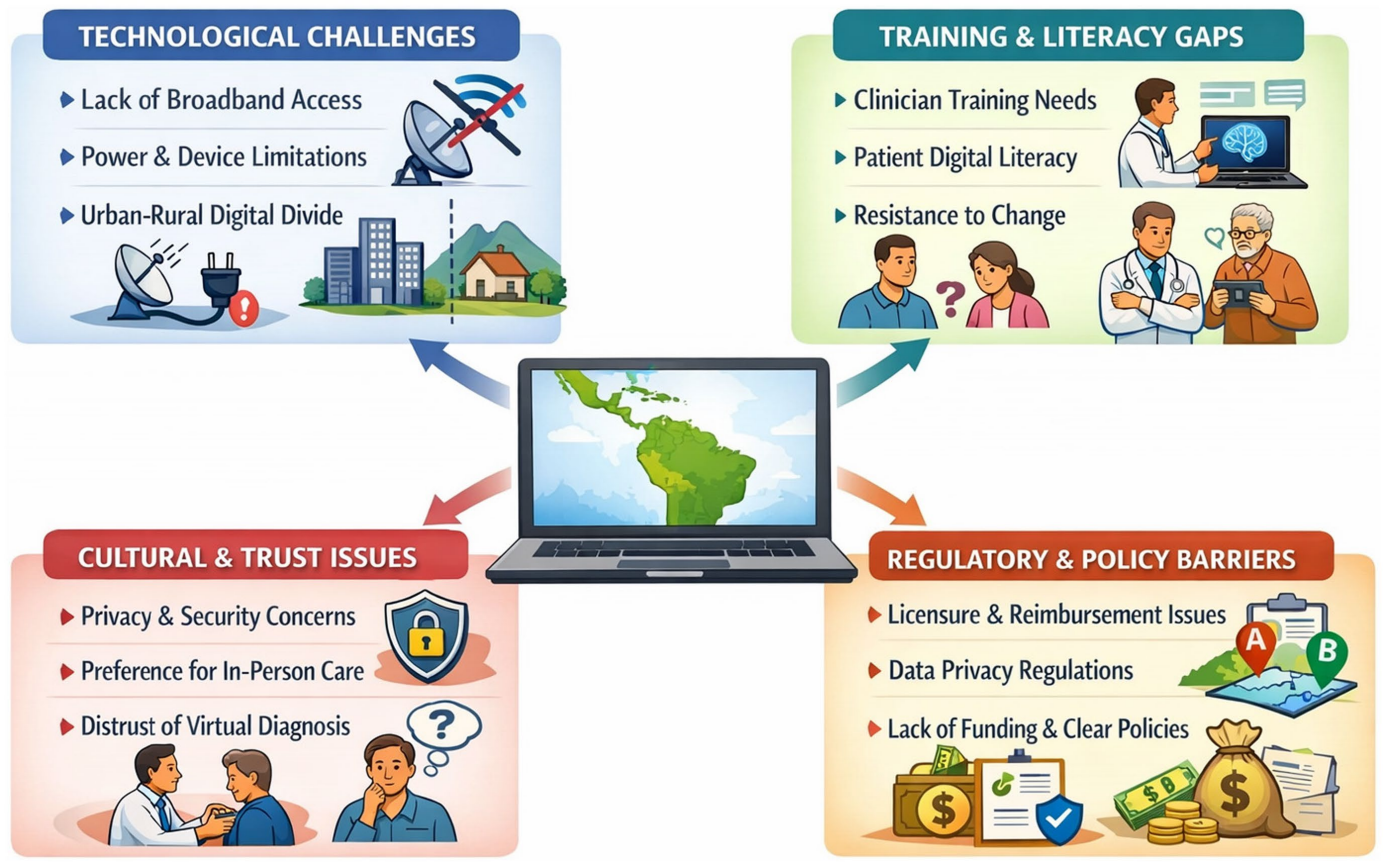
Key barriers to the implementation of teleneurology in Latin America.

While tele-neurology holds great promise, several barriers must be addressed to fully integrate these digital solutions into Latin American health systems (Figure 1).

Technological infrastructure remains a fundamental challenge given that many rural or low-income areas lack reliable broadband internet, stable electricity, or appropriate devices for telemedicine (20). Tele-neurology often requires video quality sufficient to observe subtle examination cues (like a patient’s limb movements or speech); poor connectivity can undermine the effectiveness of a virtual consult. Even within middle-income countries, the urban–rural digital divide is significant; for instance, broadband penetration and smartphone access drop sharply in remote regions, limiting who can benefit from tele-health (43). Expanding infrastructure, such as via national broadband programs or subsidized internet for health centers, is critical to ensure tele-neurology is not confined to better-off communities. Beyond infrastructure, human and organizational factors present barriers. Healthcare providers and patients both need training and familiarity with telemedicine tools; many clinicians were not formally taught how to conduct a neurological exam over video or how to use telehealth software securely (44). Likewise, patients (especially older adults or those with low education) may have limited digital literacy, making it challenging to navigate video-call platforms or patient portals (44). Dedicated training programs are needed, all medical staff should receive education on using telemedicine technologies and communicating effectively with patients online, and patients may require orientation (or assistance from community health workers) to engage in tele-consults. Without such training, tele-neurology services might be underutilized or not delivered at a high quality, which can increase medical error. Additionally, resistance to change and cultural factors can impede adoption; some providers remain concerned that virtual exams are inherently inferior to in-person visits, particularly for complex neurological assessments, which can slow their willingness to offer tele-consults (25, 44). Patients, on the other hand, might distrust a diagnosis given without “laying on of hands” or have privacy concerns about discussing sensitive issues online (25, 44). Addressing these psychosocial barriers will require demonstrating tele-neurology’s efficacy and ensuring that virtual care is patient-friendly and culturally sensitive.

Regulatory and policy barriers are also significant in the Latin American context. Telemedicine regulations in the region have historically been inconsistent or under-developed (45). Questions around medical licensure (can a neurologist licensed in Country A see patients via telemedicine in Country B or even across provincial lines?), reimbursement for telemedicine services, and data privacy laws have sometimes affected the expansion of tele-health. During the COVID-19 emergency, many governments issued temporary waivers or guidelines, but making these frameworks permanent is a work in progress. The Pan American Health Organization has noted that clear governance and legal frameworks are needed to consolidate telehealth expansion (46). In practical terms, this means updating health regulations to explicitly recognize telemedicine consultations as valid medical acts, setting standards for patient data security in virtual care, and resolving liability issues (e.g., in case of a missed diagnosis over telemedicine, how is responsibility determined?). While international tele-neurology models, including cross-border direct patient assessment, have been previously described, their large-scale implementation remains constrained by significant medico-legal challenges. These include physician licensure across jurisdictions, professional liability in the event of adverse outcomes, data protection and privacy regulations, and uncertainty regarding clinical accountability when care is delivered across national borders. Such regulatory fragmentation represents one of the most substantial barriers to international tele-neurology expansion, particularly in Latin America, where legal frameworks for telemedicine remain heterogeneous and, in some cases, underdeveloped. Addressing these limitations will require coordinated cross-country policy initiatives, regional agreements on licensure recognition, and harmonized standards for data governance and clinical responsibility. Furthermore, sustainable financing is a concern; without reimbursement mechanisms (through national insurance or health ministry budgets) that pay for tele-neurology encounters, providers may not continue offering them. Many Latin American countries are still developing telemedicine payment policies, if tele-neurology is seen as “unfunded extra work,” its growth will stall. Adequate funding is also required for equipment maintenance and technical support so that tele-neurology platforms remain functional. Certainly, the lack of an enabling policy environment, including clear national telemedicine strategies, regulatory frameworks, and budget allocation, stands as a major barrier that must be overcome to move tele-neurology from pilot projects to an integral part of health systems (46).

## 5 Discussion: opportunities and the path forward

Despite the challenges, there are substantial opportunities to take advantage of tele-neurology as a tool for health equity in Latin America. One key opportunity lies in task-sharing and integration with primary care. Given the scarcity of neurologists, tele-neurology can support general practitioners, internists, and other healthcare workers to deliver a large portion of neurological care. By using tele-consultation for specialist input, a general doctor in a remote clinic could, with the proper policies and guidelines in place, manage conditions like epilepsy or stroke stabilization that previously would have required referral (or gone untreated). The World Health Organization’s global action plan for neurological disorders emphasizes strengthening the role of primary care and leveraging digital health to enable task-sharing with specialist support (47). In practice, this could mean training primary care physicians in basic neurology and establishing tele-neurology back-up; for example, a rural doctor evaluates a patient with chronic headaches and consults a neurologist via teleconference for guidance on diagnosis and management. This model extends care to the community level and avoids over-reliance on a few tertiary centers. Early evidence suggests that such approaches are feasible, as noted, telementoring initiatives like ECHO have already shown that primary care providers, when supported remotely by specialists, can effectively handle many neurological cases (30). Expanding these networks and creating formal tele-neurology referral pathways (similar to “neurology support hotlines” for clinicians) could dramatically increase the reach of existing neurologists; it allows the limited specialist pool to multiply its impact, instead of seeing one patient at a time, a neurologist on a tele-health network can indirectly care for dozens by advising frontline providers.

Another opportunity is the widespread adoption of mobile technology and apps to complement formal telemedicine services. Latin America has high mobile phone penetration, and even in lower-income groups, access to a basic smartphone is increasingly common. This opens the door for mHealth strategies in neurology; for instance, simple smartphone applications can assist patients in tracking their symptoms (seizure logs, migraine diaries) and share that data with doctors remotely. Apps with built-in educational content (in local languages) can improve patient understanding of neurological conditions and adherence to treatment, as well as identification of warning sign or red flags to seek prompt evaluation and treatment. Additionally, a neurologist could follow up with a patient by reviewing blood pressure readings or medication side effects over WhatsApp in between formal visits; while not a substitute for comprehensive care, these low-cost, accessible tools can strengthen the continuity of neurological care, especially when travel is difficult and road accessibility is scarce. Governments and health organizations have the opportunity to develop or endorse secure messaging platforms and apps tailored to healthcare, so that providers can use them with confidence in privacy and reliability, following the statues of each countries’ public health ministry or regulatory authority. There is also potential for SMS-based systems to deliver public health messages or reminders (for example, reminding patients to refill their anti-seizure medications or attend a teleclinic); embracing mobile health could significantly extend tele-neurology’s reach to patients who might not engage with traditional telemedicine due to technology barriers as SMS is more universally accessible than a video call requiring broadband.

AI and emerging technologies present further opportunities to enhance tele-neurology services as it could be a force multiplier for the limited specialist workforce; for example, machine learning algorithms are being developed to interpret EEG results or identify abnormalities on brain MRI scans (41). Integrating these tools into telemedicine platforms could allow a preliminary automated analysis that alerts the remote neurologist to areas of concern, or even helps non-specialist providers make interim decisions. AI-driven clinical decision support could guide primary care physicians through neurological exam steps or suggest possible diagnoses based on patient inputs. Importantly, AI can also help manage the administrative load; recent innovations include AI systems that automatically generate clinical visit notes from telemedicine video calls, which could save neurologists time on documentation and let them see more patients. Latin American innovators and governments are increasingly recognizing these possibilities as countries invest more and more in digital health, ensuring that AI tools are introduced in an equitable manner (with appropriate training and safeguards). Moreover, technologies like remote monitoring devices, wearable sensors, and even virtual reality could be leveraged for neurological care. Examples of remote monitoring already exists such as wearable seizure alarms that notify caregivers or telemedicine centers when a patient has a convulsive seizure, or home sensors that track mobility in Parkinson’s disease (37, 38). These technologies generate data that can be reviewed via telemedicine, enabling proactive interventions (for example, adjusting therapy if a patient’s gait worsens or seizures increase). As costs of devices come down, Latin America stands to benefit from these innovations, particularly if international partnerships and knowledge exchange make new tools more accessible.

Finally, the momentum behind regional cooperation and networks is a significant opportunity. The challenges of neurological care are shared across countries, and tele-neurology provides a platform for cross-border collaboration. I see this in initiatives like the Latin American virtual tumor board for neurosurgery (31); such collaborations can be expanded to other areas, for example, a Latin American teleneurology alliance could allow neurologists in one country to periodically consult on difficult cases in another country that has fewer specialists. Regional professional societies (e.g., the Latin American Federation of Neurological Societies) could host regular tele-case conferences or continuing education webinars, which not only improve care but also foster a sense of community and shared mission. Tele-neurology thus can break down the traditional silo of care within national borders, allowing expertise to flow where it’s needed. This aligns with the Pan American Health Organization’s vision of leveraging digital health to improve regional health solidarity; by working together through telemedicine platforms, countries can also collectively negotiate better terms for telemedicine technologies, share best practices in regulation, and perhaps develop standardized protocols suited to Latin American contexts. In essence, tele-neurology does not just connect doctors to patients, it can connect health systems to each other, creating a neurological learning health network across the continent.

Tele-neurology should therefore be deliberately integrated into national public health strategies rather than treated as a temporary or ancillary solution. Embedding tele-neurology within primary care strengthening efforts offers a pragmatic pathway to extend specialist expertise in settings where neurologists are scarce, allowing frontline clinicians to manage common neurological conditions with remote specialist support (46). Such integration aligns with global public health priorities, including the World Health Organization Intersectoral Global Action Plan on Epilepsy and Neurological Disorders (47), which emphasizes task sharing, digital health, and continuity of care as mechanisms to reduce the neurological treatment gap. For tele-neurology to be sustainable beyond the COVID 19 context, however, countries must move from pilot initiatives to structured national programs supported by stable financing, regulatory clarity, workforce training, and interoperable digital infrastructure (46). Regional collaboration further represents a strategic opportunity, as shared tele-neurology networks, cross border specialist consultations, and coordinated policy development could help overcome national workforce limitations while promoting standardization and quality of care. Encouraging signs include countries like Brazil, where telehealth services initiated in primary care have continued and expanded after 2020, and others like Colombia, which conducted national telemedicine surveys to inform long-term strategy (26, 40). Another consideration for integration is the engagement of multiple sectors and stakeholders. Tele-neurology does not only involve health ministry directives; it intersects with telecommunications (internet access), education (training curriculum for medical professionals), and finance (innovative funding or public-private partnerships for technological development). A systems-level perspective means convening stakeholders (health providers, IT specialists, patient advocacy groups, telecommunication companies, and academic institutions) to collaboratively develop tele-neurology initiatives. One successful example is in Mexico, where a collaboration between the national health system and a technology NGO created a telemedicine platform for remote areas, drawing on each sector’s strengths. Similarly, academic neurology departments in the region (e.g., in Argentina and Colombia) have played key roles in running telemedicine pilot projects and evaluating their impact; involving them in policy design can ensure that systems are evidence-based and iteratively improved. Ultimately, tele-neurology should be viewed not as a replacement for in person services, but as a core component of a hybrid care model that improves equity, efficiency, and resilience of neurological care systems across Latin America.

## 6 Conclusion

Tele-neurology in Latin America has evolved from a niche innovation into a practical strategy to address longstanding gaps in neurological care. The COVID-19 pandemic demonstrated that rapid digital transformation is feasible and, when supported by policy and investment, can be sustained beyond crisis conditions. Experiences across the region show that tele-neurology can meaningfully expand specialist access, whether through video consultations in rural clinics, smartphone-based follow-up, or remote interpretation of diagnostic data; these approaches are not technology-driven ends in themselves but tools to reduce inequities and extend care to populations historically excluded from specialist neurology services. The central challenge now is no longer feasibility, but optimization and scale. Sustainable financing, appropriate training and accreditation, data protection, and equitable access must guide the integration of tele-neurology into routine health systems. While there is no single model suitable for all countries, shared priorities emerge, including investment in digital infrastructure and literacy, regional collaboration, patient-centered service design, and continuous evaluation using locally generated evidence. Tele-neurology should therefore be viewed as a core component of a broader strategy to reduce the neurological treatment gap in Latin America. Although it cannot replace parallel efforts in prevention, pharmacological access, and rehabilitation, digital solutions offer a powerful means to accelerate progress toward more equitable neurological care. Realizing this potential will require sustained political commitment, sound policy design, and cross-sector collaboration to ensure that geography and workforce shortages no longer determine neurological outcomes in the region.
